# Cross-Talk between PPAR*γ* and Insulin Signaling and Modulation of Insulin Sensitivity

**DOI:** 10.1155/2009/818945

**Published:** 2010-02-23

**Authors:** Anna Leonardini, Luigi Laviola, Sebastio Perrini, Annalisa Natalicchio, Francesco Giorgino

**Affiliations:** Section of Internal Medicine, Endocrinology, Andrology and Metabolic Diseases, Department of Emergency and Organ Transplantation, University of Bari School of Medicine, Piazza Giulio Cesare, 11, 70124 Bari, Italy

## Abstract

PPAR*γ* activation in type 2 diabetic patients results in a marked improvement in insulin and glucose parameters, resulting from an improvement of whole-body insulin sensitivity. Adipose tissue is the major mediator of PPAR*γ* action on insulin sensitivity. PPAR*γ* activation in mature adipocytes induces the expression of a number of genes involved in the insulin signaling cascade, thereby improving insulin sensitivity. PPAR*γ* is the master regulator of adipogenesis, thereby stimulating the production of small insulin-sensitive adipocytes. In addition to its importance in adipogenesis, PPAR*γ* plays an important role in regulating lipid, metabolism in mature adipocytes by increasing fatty acid trapping. Finally, adipose tissue produces several cytokines that regulate energy homeostasis, lipid and glucose metabolism. Disturbances in the production of these factors may contribute to metabolic abnormalities, and PPAR*γ* activation is also associated with beneficial effects on expression and secretion of a whole range of cytokines.

## 1. Introduction

As a major tissue for whole-body energy homeostasis, adipose tissue integrates both central and peripheral metabolic signals that orchestrate energy balance. An imbalance between energy intake and energy expenditure leads to the expansion of adipose tissue, characterized by a combination of cell proliferation (hyperplasia) and cell size increase (hypertrophy). A complex and yet incompletely defined series of transcriptional events represents the fundamental biological mechanism through which multipotent mesenchymal precursor cells become committed to the adipocyte lineage and exhibit the typical markers of mature fat cells. Identifying the mechanisms that control differentiation of adipose cells would provide clues for designing comprehensive therapeutic strategies for the prevention and treatment of adipose tissue expansion and its associated clinical disorders, including hyperlipemia, hypertension, and type 2 diabetes. However, the mechanisms that regulate adipose cell number and size during adipogenesis are still poorly understood.

In recent years, it has become evident that the societies of the developed countries are at immense risk of metabolic diseases, the so-called civilization diseases or X syndrome. In fact, the rise in the prevalence of specific endocrine-related diseases such as obesity and diabetes clearly suggests an importance of either environmental or genetic factors. The therapy of metabolic diseases assumes the recognition and detailed understanding of the molecular events that control these disorders as well as the development of therapeutics targeting the responsible factors. Recently, several different transcriptional factors have been identified as regulators of the expression of a set of genes involved in glucose and lipid metabolism. Among them, peroxisome proliferator-activated receptors (PPARs), belonging to the superfamily of nuclear receptors (NRs), have been shown to play a central role in the transcriptional control of genes encoding proteins involved in the above processes.

## 2. PPAR Nuclear Receptors

**Figure 1:**
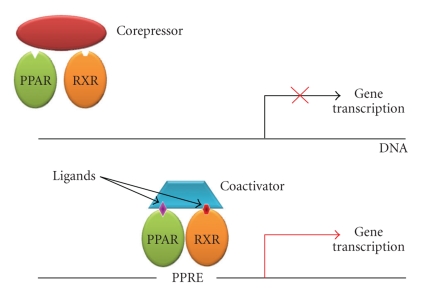
Mechanism of PPAR*γ* activation. Upon ligand binding to the PPAR/RXR heterodimer, a conformational change leads to release of a corepressor and binding of a coactivator; this regulates the kinetics of the assembly of the transcription complex, resulting in increased affinity for the specific PPAR response element, which modulates gene transcription. RXR; Retinoic X receptor; PPRE; PPAR response element.

Peroxisome proliferator-activated receptors (PPARs) exist as an obligate heterodimer with the retinoic X receptor (RXR) [1] and are localized to the nucleus also in the unligated state [2]. Upon ligand binding, a conformational change leads to corepressor release and coactivator binding. The binding pocket permits binding of ligands with quite diverse structures [3], probably resulting in different conformational changes which, in turn, affect the recruitment of cofactors and regulate the kinetics of the assembly of the transcription complex, as well as the affinity for the specific PPAR response element (PPRE). The PPAR/RXR heterodimers can be activated by ligands of either receptor, and simultaneous binding of both ligands has been shown to be more efficient in some cases [4]. After ligand binding and activation, the heterodimers are able to either enhance or repress gene expression through binding to PPRE in the promoter region of target genes (Figure 1).

Three different human PPAR subtypes have been identified so far, designated as PPAR*α*, PPAR*β* (also known as PPAR*δ*), and PPAR*γ*. Each of them displays a distinct pattern of tissue distribution and a specific role. PPAR*α* is predominantly expressed in the liver and skeletal muscles, participating in fatty-acids catabolism. PPAR*α* also activates fatty-acid oxidation in the kidney, skeletal muscles, and heart [5]. It has been established that PPAR*β* is present at moderate levels in all human tissues, with a higher expression in the placenta and the large intestine [6]. Very little is known about the functions of PPAR*β*. However, recent findings have implicated PPAR*β* as an important regulator of energy expenditure as well as glucose and lipid metabolism [7]. Of the three members of PPARs, PPAR*γ* is the most frequently studied nuclear receptor involved in the control of energy balance and both lipid and glucose homeostasis [8]. PPAR*γ* exists as two protein isoforms, PPAR*γ*1 and *γ*2, that differ in their N-terminal end as a result of alternative promoter usage [8]. PPAR*γ*1 has a similar expression pattern as PPAR*α* while PPAR*γ*2 is predominantly expressed in adipose tissue where it regulates adipocyte differentiation.

## 3. Endogenous and Synthetic Ligands

Over the past several years, various natural and synthetic PPAR*γ* ligands, including PPAR*γ* agonists, PPAR*γ* partial agonists, and PPAR*α*/*γ* dual agonists, have been investigated. Numerous studies have shown that polyunsaturated fatty acids and related molecules can activate PPAR*γ* as well as other PPARs [9–11]. Interestingly, PPAR*γ* responds poorly to native fatty acids compared to PPAR*α* and PPAR*δ*, suggesting that modified fatty acids may be the biological ligands. Certain prostanoids, including 15-deoxy-Δ12,14 prostaglandin J2 (15-dPGJ2), are excellent activators of PPAR*γ* [12, 13]. However, it is unlikely that 15-dPGJ2 is present at sufficient levels in vivo to be a biologically significant ligand. Oxidized fatty acids, such as 9-hydroxy-10,12-octadecadienoic acid and 13-hydroxy-9,11-octadecadienoic acid found in oxidized low-density lipoprotein (LDL), activate PPAR*γ* with increased potency and efficacy relative to native fatty acids and are present at significant concentrations in atherosclerotic lesions [14]. Whether oxidized fatty acids serve as activators in other tissues, however, is not clear. It is possible that different ligands for PPAR*γ* may be of primary importance in other contexts. For example, the ligand responsible for PPAR*γ* activation in adipogenesis may be distinct from those that activate PPAR*γ* in macrophages in the artery wall. Other lipids, such as nitrated fatty acids and lysophosphatidic acid, have also been reported to activate PPAR*γ* [15, 16]. The importance of these molecules in PPAR*γ* biology remains to be established. 

The synthetic PPAR*γ* agonists are thought to be factors determining adipocyte differentiation as well as potential antidiabetic drugs [17]. Compounds such as glitazones or thiazolidinediones (TZDs) (pioglitazone and rosiglitazone) are used clinically as insulin sensitizers [18]. They activate PPAR*γ* and decrease insulin resistance and glucose level in the serum of patients with type 2 diabetes [18]. Many drugs belonging to the TZD class exhibit high selectivity for PPAR*γ* and minimal or no activity toward subtypes-*α* and -*β* [19]. However, despite significant antidiabetic activities, TZDs may cause several side effects, such as increased adiposity, oedema, and an increased rate of fractures of the small bones of the extremities. From the therapeutic point of view, improvement of the pharmacological profiles of PPAR*γ* ligands is highly required. Therefore, an alternative approach, relying on the identification of partial agonists, was developed. It was recently reported that a PPAR*γ* partial agonist similar to LSN862, that is, (S)-2 methoxy-3-{4-[5-(4-phenoxy)pent-1-ynyl]phenyl}-propionic acid, has better antidiabetic activity and weaker side effects than the TZDs [20]. More recently, a novel family of PPAR*γ* partial agonists (pyrazol-5-yl benzenesulfonamide derivatives) with either high potency or specificity in vitro or glucose-lowering efficacy in vivo has been identified [21]. Interestingly, the X-ray structures of the PPAR*γ*-ligand complexes revealed a lack of hydrogen bonds between them. This is in sharp contrast to PPAR*γ* agonists sharing a common binding mode in which the acidic head groups form a network of hydrogen interactions with His-323, His-449, and Tyr-473 within the ligand binding pocket [22]. Further molecular studies are required to understand how PPAR*γ* partial agonists modulate transcriptional activity through the recruitment of coactivator and corepressor proteins.

Recent discoveries point to ligands that could stimulate more than one isotype of PPAR at similar concentrations. Thus, the insulin-sensitizing effects of PPAR*γ* and the anti-dislipidemic effects of PPAR*α* or *β* can be simultaneously obtained by using the so-called coligands. PPAR*α*/*γ* coligands (ragaglitazar, O-arylmandelic acid, LY465608, and KRP-297) have been shown to have better insulin-sensitizing and lipid-lowering potential in diabetic rodents, as compared to standard compounds which can only stimulate one isotype of PPAR [23–25].

## 4. PPAR*γ* and Insulin Signaling

PPAR*γ* activation through binding of the synthetic TZDs in type 2 diabetic patients results in a marked improvement in whole-body insulin sensitivity, leading to reduced insulin and glucose plasma levels. The mechanisms of PPAR*γ*-mediated insulin sensitization are complex and are thought to involve specific effects on fat, skeletal muscle, and liver, even though adipose tissue appears to be the major target of TZD-mediated effects on insulin sensitivity. At the cellular level, PPAR*γ* activation has been shown to affect the insulin signaling cascade, through direct modulatory effects on the expression and/or phosphorylation of specific signaling molecules.

**Figure 2:**
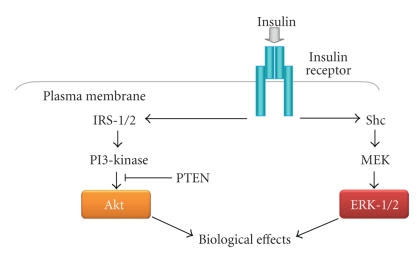
Insulin signaling pathway in adipose cells. Binding of insulin to its tyrosine kinase receptor engages a cascade of intracellular phosphorylation events, including activation of phosphatidylinositol-3-kinase and ERK-1/2, that promote multiple biological responses, including glucose uptake, lipid metabolism, survival, differentiation, and modulation of gene transcription.

**Figure 3:**
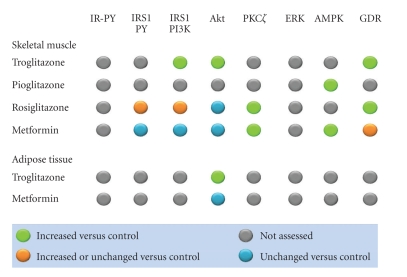
Effects of TZDs and metformin on activation of insulin signaling proteins in tissues from individuals with type 2 diabetes. The effects of troglitazone, pioglitazone, and rosiglitazone on various proteins involved in insulin signaling in skeletal muscle and adipose tissue are indicated. The effects of metformin are also shown for comparison. GDR indicates the glucose disposal rate, as a measure of insulin sensitivity. IR-PY: insulin receptor tyrosine phosphorylation; IRS1: insulin receptor substrate-1; PY: tyrosine phosphorylation; PI3K: phosphatidylinositol 3 kinase. Adapted from [26–33].

Binding of insulin to its tyrosine kinase receptor engages a cascade of intracellular phosphorylation events, including tyrosine phosphorylation of insulin receptor substrate (IRS) proteins and activation of phosphatidylinositol-3-kinase (PI 3-kinase) and other downstream kinases, which promote multiple biological responses, including glucose uptake, lipid metabolism, survival, differentiation, and modulation of gene transcription (Figure 2). Several groups have shown that PPAR*γ* activation can influence insulin signaling at various steps in these pathways, resulting in improved whole-body insulin sensitivity and enhanced glucose and lipid metabolism. The effects of TZDs on activation of insulin signaling proteins in skeletal muscle and adipose tissue from individuals with type 2 diabetes are summarized in Figure 3.

### 4.1. IRS Proteins

The IRSs are a large family of docking proteins that act as an interface between the insulin receptor and a complex network of intracellular-signaling molecules. Hammarstedt et al. [34] observed no change in the expression of multiple insulin signaling molecules, including IRS-1, in adipose tissue of pioglitazone-treated nonobese, insulin-resistant individuals [35]. However, a number of studies have demonstrated modulatory effects of TZDs on IRS phosphorylation. In both HEK-293 cells overexpressing a recombinant IRS-1 protein and 3T3-L1 adipocytes, rosiglitazone reduces the PMA-induced inhibitory serine phosphorylation of IRS-1 and restores downstream insulin signaling [36]. The increased levels of IRS-1 serine phosphorylation seen in adipose cells of obese Zucker rats were also found to be reduced after TZD treatment. TZDs may act primarily by reducing the circulating levels of FFA, which have been shown to induce serine phosphorylation of IRS-1 through activation of the protein kinase C isoform PKC*θ* [37]. In obese Zucker rats, short-term treatment with both rosiglitazone and a non-TZD PPAR*γ* ligand could potentiate the insulin effect and increase the tyrosine phosphorylation of the insulin receptor and IRS-1 as well as induce activation of Akt/PKB [38]. Effects of PPAR*γ* activation have also been reported on IRS-2: in both cultured human adipocytes and 3T3-L1 adipocytes, IRS-2 was found to be increased, both at the gene and protein level, after pioglitazone treatment [39].

### 4.2. The PI 3-Kinase/Akt Pathway

PI-3 kinase acts as a critical signaling molecule triggering a number of insulin-stimulated effects, including glucose uptake, glycogen synthesis, and cell differentiation. Multiple clinical studies have investigated the effects of TZDs on glucose disposal rates and the insulin signal transduction system in type 2 diabetic patients. TZDs, particularly troglitazone and rosiglitazone, were found to markedly improve glucose disposal rates [26, 27], whereas the effects of metformin appeared less prominent [28, 29]. Studies in which biopsies of subcutaneous abdominal adipose tissue of diabetic patients were taken before and after a period of therapy with either metformin or troglitazone showed no significant effects on total cellular levels of p85, p110*β*, or Akt proteins with either treatment; however, the insulin effect on Akt phosphorylation was increased with troglitazone, while it was unaltered after metformin treatment [30]. The effects of TZDs on insulin signaling molecules have also been investigated in human skeletal muscle. Treatment with troglitazone increased insulin-stimulated IRS-1-associated PI 3-kinase activity and Akt activity in skeletal muscle biopsies from type 2 diabetic patients [26] and enhanced Akt phosphorylation in skeletal muscle from glucose-tolerant, insulin-resistant, first-degree relatives of type 2 diabetic patients [31]. More controversial appear to be the effects of rosiglitazone on PI 3-kinase activity and Akt phosphorylation. While Miyazaki et al. showed that the improvement in insulin-stimulated muscle glucose disposal after rosiglitazone therapy was associated with increased IRS-1 tyrosine phosphorylation and IRS-1-associated PI 3-kinase activity [32], Karlsson et al. found no changes in IRS-1/PI 3-kinase and Akt/AS160 signaling in patients with newly diagnosed type 2 diabetes, thus concluding that the insulin-sensitizing effects of rosiglitazone were independent of enhanced insulin signaling via these proteins [28]. Interestingly, no effect of metformin therapy on PI 3-kinase or Akt activation in diabetic muscle has been documented [26, 29].

### 4.3. 5′-AMP-Activated Protein Kinase (5′-AMP Kinase)

**Figure 4:**
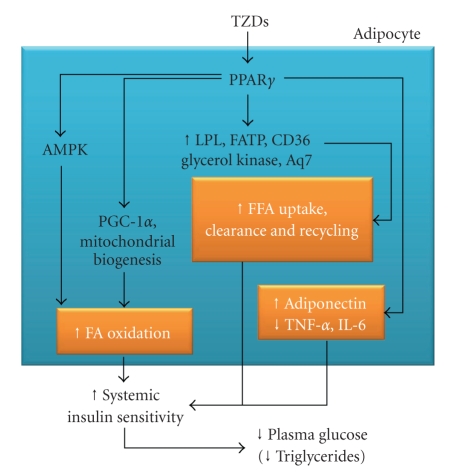
Cellular effects of PPAR*γ* activation in adipocytes. TZDs improve whole-body insulin sensitivity by modulating glucose and lipid metabolism in adipose tissue as well as adipokine secretion by adipocytes. FA: fatty acids.

5′-AMP kinase is a key regulator of both glucose and lipid metabolism, which is associated with improved insulin signaling and enhanced insulin sensitivity in skeletal muscle. 5′-AMP kinase activation increases fatty acid oxidation in skeletal muscle by decreasing malonyl CoA concentrations. Both TZDs (i.e., pioglitazone) [33] and metformin [29] have been shown to improve glucose tolerance via adenosine 5′-AMP kinase. Activation of AMPK by metformin decreased the level of plasma glucose and plasma triglycerides by promoting muscle glucose uptake and inhibiting hepatic glucose output [40]. Recently, Coletta et al. have demonstrated that pioglitazone activates 5′-AMP kinase and acetyl-CoA carboxylase (ACC) in human muscle biopsies from patients with type 2 diabetes, leading to increased expression of genes involved in mitochondrial function and fat oxidation, and reduced toxic burden of intracellular lipid metabolites (fatty acyl CoA, diacylglycerol, ceramides) [33] (Figure 4).

### 4.4. ERK-1/2

The ERK proteins, which belong to the family of MAP kinases, modulate cellular responses to environmental stress, cell survival, proliferation, and differentiation. Transfection of cultured cells with a dominant negative MEK, which is the ERK activating kinase, results in decreased effects of both insulin and TZDs on PPAR*γ* activity, suggesting that ERK is involved in the cross-talk between insulin and PPAR*γ* [41]. In vitro assays demonstrate that both ERK2 and JNK are able to phosphorylate PPAR*γ*2 [42]. The MAPK phosphorylation site, which can be used by both ERK- and JNK-MAPK [43], was mapped at serine 82 of mouse PPAR*γ*1, which corresponds to serine 112 of mouse PPAR*γ*2 [44]. Substitution of this serine by alanine (S82A) leads to a loss of PDGF-mediated repression of PPAR*γ* activity [45]. Human PPAR*γ*1 phosphorylation at this site (S84) inhibits both its ligand-dependent and ligand-independent transactivating function. The S84A mutant showed an increase in the AF-1 transcriptional activity of PPAR*γ* [46]. Treatment of macrophages with TGF*β*1 increases PPAR*γ* phosphorylation and decreases TZD-induced CD36 expression via activation of the ERK-MAPK pathway [47]. Mutation of the main MAPK site of phosphorylation in PPAR*γ*2 (S112D) results in a decreased ligand-binding affinity [41]. Limited protease digestion shows that the unliganded PPAR*γ*2 and the S112D mutant have different sensitivity; thus, the phosphorylation status of serine 112 plays a role in the conformation of the unliganded receptor which regulates the affinity of PPAR*γ* for its ligands and affects its coactivator recruitment ability [44]. It has been proposed that phosphorylation-mediated inhibition of transcriptional activity of nuclear receptors is an important “off-switch” of ligand-induced activity (reviewed in [48]). Extracellular signals which activate intracellular phosphorylation pathways can also influence the degradation process of PPAR*γ* [49]. As an example, treatment of cells with an MEK inhibitor blocks the degradation of PPAR*γ*. However, not all phosphorylation events are inhibitory and enhance proteosomal degradation, and thus care must be taken when making a global speculation. Substitution of proline to glutamine at position 115 renders PPAR*γ* constitutively active through the modulation of the MAPK-dependent phosphorylation status of serine 114 [50]. Subjects carrying this mutation are extremely obese but surprisingly show a lesser insulin resistance than expected. Mice homozygous for the S112A mutant (homologous to human S114) [51] are protected against diet-induced obesity. This may be due to changes in adipocyte function, such as secretion of adiponectin and leptin. Overall, prevention of PPAR*γ* phosphorylation leads to an improvement of insulin sensitivity mainly due to increased glucose disposal in muscle, which is similar to TZD treatment [51].

### 4.5. PPAR*γ* and the Glucose Transport System

PPAR-*γ* activity has been shown to directly regulate the expression of GLUT4 [52] and c-Cbl associating protein (CAP), both involved in regulating insulin-stimulated glucose transport [53]. The GLUT4 (insulin-dependent) transporter is a key modulator of glucose disposal in both muscle and fat. TZD treatment increased the expression of the insulin-responsive glucose transporter GLUT4. However, in another report of the effect of rosiglitazone on freshly isolated human adipocytes, no effect could be seen on the expression of GLUT4 [54]. In animal models of obesity and diabetes, in which the expression of GLUT4 in adipose cells is reduced, treatment with troglitazone restored its expression to normal levels [55]. Although no complete PPRE has been found in the GLUT4 promoter, PPAR*γ* and its heterodimer partner RXR*α* have been found to bind and repress the promoter activity of GLUT4. The repression is augmented in the presence of the natural ligand, 15D-prostaglandin J2, but completely alleviated by rosiglitazone [56]. This is a novel mechanism through which a PPAR*γ* ligand can exert an antidiabetic effect, that is, by detaching the PPAR*γ* transcription complex from the promoter, thereby increasing the expression of the target gene. It has also been demonstrated that TZDs increase the expression of CAP either in 3T3-L1 adipocytes and in Zucker (fa/fa) diabetic rats, resulting in the stimulation of glucose transport [57]. The induction of CAP expression by TZDs takes place through direct binding of activated PPAR-*γ*/RXR*α* heterodimers to a PPRE in the CAP promoter [53].

Interestingly, experimental deletion of PPAR*γ* results in a decrease in insulin-stimulated glucose transport into 3T3-L1 adipocytes, and this is due to a decrease in GLUT1 and GLUT4 function [58]. It remains to be investigated, however, whether similar direct effects on glucose uptake are also operating in skeletal muscle, where much lower levels of PPAR*γ* expression are observed, but where the majority of glucose disposal occurs. Unfortunately, conflicting findings in the two existing mouse models of muscle-specific PPAR*γ* deletion have so far failed to resolve this issue [59, 60] (see below).

The intracellular protein PTEN (phosphatase and tensin homolog deleted on chromosome 10) has been recently proposed to play a crucial role in the PPAR*γ*-induced regulation of glucose uptake. Kim et al. have demonstrated that the reduction of PTEN expression in skeletal muscle cells and adipocytes may be a primary mechanism of the PPAR*γ*-induced improvement in glucose uptake. Furthermore, decreased PTEN levels, associated with reduced plasma glucose, were observed in adipose and muscle tissues of *ob/ob* mice treated with two structurally different PPAR*γ* agonists, thus confirming that PPAR*γ*-induced insulin sensitization in vivo is mediated by PTEN downregulation [61].

Several lines of evidence support an emerging role for PPAR*δ* in muscle for glucose and lipid metabolism. The role of PPAR*δ* on whole-body glucose homeostasis has been evaluated in muscle-specific PPAR*δ* transgenic mice [62], which exhibit enzymatic and gene expression profiles that promote oxidative metabolism in skeletal muscle. Moreover, PPAR*δ* transgenic mice have reduced body fat mass due to a reduction of adipose cell size [63]. Given the importance of skeletal muscle insulin resistance in the development of type 2 diabetes and other metabolic diseases, targeted activation of PPAR*δ* in skeletal muscle may represent a novel therapeutical target to enhance glucose metabolism. Indeed, there is evidence that exposure of primary human skeletal muscle cells and C2C12 mouse myotubes to specific PPAR*δ* agonists enhances basal and insulin-stimulated glucose uptake [64]. 

## 5. Tissue-Specific PPAR*γ* Effects

### 5.1. Adipose Tissue

PPAR*γ* has the highest expression levels in adipose tissue compared with other metabolic organs, such as skeletal muscle, liver, and pancreas. PPAR*γ* activation in mature adipocytes induces the expression of a number of genes involved in the insulin signaling cascade, thereby improving insulin sensitivity. PPAR*γ* is the master regulator of adipogenesis, thereby stimulating the production of small insulin-sensitive adipocytes. In addition to its importance in adipogenesis, PPAR*γ* plays an important role in regulating lipid metabolism in mature adipocytes. The induction of adipogenesis associated with the capability for fatty acid trapping has been shown to be an important contributor to the maintenance of systemic insulin sensitivity. Finally, adipose tissue produces several hormones that regulate energy homeostasis, lipid, and glucose metabolism such as leptin, adiponectin, resistin, tumor necrosis factor-*α*, and others. Disturbances in the production of these factors may contribute to the development of insulin resistance or impaired insulin secretion: PPAR*γ* activation is also associated with beneficial effects on the expression and secretion of a whole range of adipokines. 

#### 5.1.1. The Role of PPAR*γ* in Adipogenesis and Differentiation.

Adipogenesis refers to the differentiation process of preadipocyte precursor cells into mature adipocytes during which gene expression, cell morphology, and hormone sensitivity change. Preadipocytes can be differentiated into white (energy storage) and brown (energy dissipation) adipocytes. In the differentiation of white adipocytes, numerous genes encoding proteins participating in fatty-acid metabolism are induced. It is known that the transcription factor PPAR*γ* is an important regulator of the formation of adipose tissue [65–69], since it induces several specific adipose markers, such as adipocyte fatty acid binding protein (aP2) [70], phosphoenolpyruvate carboxykinase (PEPCK) [71], and lipoprotein lipase (LPL) [72]. Moreover, the ectopic expression of PPAR*γ* promotes adipogenesis in nonadipogenic fibroblastic cells such as NIH-3T3 cells [73]. In addition, PPAR*γ*-deficient adipocytes of adult mice die within a few days [73] and PPAR*γ* knockout mice are unable to develop adipose tissue [8]. Consistent with the above, several PPAR*γ* missense mutations (C190S, V290M, F388L, R425C, P467L) in humans are associated with partial lipodystrophy [74]. Although all these studies indicate a pivotal role of PPAR*γ* in adipogenesis, it is likely one of several proteins involved in the regulation of this multifactoral process. Indeed, besides PPAR*γ*, C/EBP transcription factors (C/EBP-*α*, -*β*, and -*δ*) expressed in distinct phases of adipogenesis have been shown to play important roles as well. C/EBP-*β* and -*δ* are activated in response to insulin or glucocorticoids in the initial stages of adipogenesis [75, 76] and they, in turn, induce the transcription of PPAR*γ*. 

With cell differentiation, mRNA levels of PPAR*γ* are markedly upregulated [77]. In addition, during the early stages of differentiation, another transcriptional factor, namely, ADD1/SREBP1, has been found to affect the transcriptional activity of PPAR*γ* [78]. It has been suggested that this factor can modulate PPAR*γ* activity through the production of endogenous ligands for PPAR*γ* since it participates in the regulation of cholesterol homeostasis and in the expressions of several genes encoding proteins involved in lipid metabolism [75]. In the terminal stages of adipogenesis, PPAR*γ* activates the expression of C/EBP-*α*; however, C/EBP-*α*, in response, also induces PPAR*γ* gene expression through binding to the same DNA sites in the PPAR*γ* promoter that are induced by C/EBP-*β*, and -*δ* [79]. Thus there is a positive feedback loop between PPAR*γ* and C/EBP-*α* [80]. The positive cross-regulation between these factors has been observed in C/EBP-*α*-deficient adipocytes, which accumulate fewer lipids and do not induce endogenous PPAR*γ* [80].

The adipogenic effect of PPAR*γ* activation is likely related to the known effects of glitazones to enhance bone loss and lead to increased risk of bone fractures, which has emerged from clinical trials. Within the bone marrow, the differentiation of the resident mesenchymal stem cells (MSCs) into adipocytes or osteoblasts is competitively balanced, so that mechanisms that promote a given cell fate (i.e., osteogenesis) actively suppress mechanisms that induce the alternative lineage (adipogenesis). Elbrecht et al. [81] first showed that PPAR*γ* is expressed in bone marrow MSCs. Subsequently, it was demonstrated that treatment of bone marrow stromal cells with TZDs resulted in the differentiation of these cells into adipocytes [82] rather than osteoblasts. It has thus been suggested that this unbalanced marrow adipogenesis may contribute to the increased risk of bone fractures in TZD-treated subjects.

In addition to the above transcription factors activating adipogenesis, there are several factors involved in the control of this process, such as tumor necrosis factor- (TNF-) *α* and leptin. TNF-*α* is a polypeptide hormone with pleiotropic effects on cellular proliferation and differentiation and is a potent inhibitor of adipogenesis. The exposure of 3T3-L1 adipocytes to TNF-*α* results in lipid depletion and a complete reversal of adipocyte differentiation [83]. In addition, suppression of several adipocyte genes, such as those encoding aP2, adipsin, and insulin-responsive glucose transporter (GLUT4), has been found [84–86]. This antiadipogenic effect of TNF-*α* most likely results from the downregulation of C/EBP-*α* and PPAR*γ* expression [87]. In the case of leptin, which induces lipolysis and glucose utilization in adipocytes, it has been shown that TZD-activated PPAR*γ* inhibits leptin production [88]. This inhibition can be explained in terms of a functional antagonism between C/EBP-*α* and PPAR*γ* on leptin promoter activity [89].

Apart from adipocyte differentiation, PPAR*γ* activation promotes the apoptosis of mature adipocytes [90]. It has been reported that troglitazone, a PPAR*γ* agonist of the TZD class, increases the population of small adipocytes in white adipose tissue and concomitantly decreases the population of large adipocytes. In addition, the percentage of apoptotic nuclei is increased by 2.5-fold in troglitazone-treated tissues, implying that large adipocytes lost by apoptosis may be counterbalanced by small adipocytes newly differentiated following troglitazone treatment. PPAR*γ* activation by TZD thus leads to the accumulation of small adipocytes, which are more insulin sensitive than the large adipocytes [90].

#### 5.1.2. Modulation of Adipokine Production

Another potential mechanism whereby activation of PPAR*γ* in adipose tissue may impact whole-body insulin sensitivity is by modulating the production of adipokines. Adiponectin is a multimeric plasma protein produced exclusively by adipose tissue that shares homology with the c1q complement protein. Multiple studies have shown that plasma adiponectin levels are inversely correlated with adipose tissue mass and directly correlated with insulin sensitivity [91]. The adiponectin gene is a direct target for regulation by PPAR*γ* [92]. Adiponectin mRNA and plasma protein levels are induced in rodents and humans following TZD administration [93, 94]. Studies in mice have shown that administration of adiponectin leads to suppression of hepatic glucose output and improvement in glucose uptake, reminiscent of the effects of TZDs [95]. Furthermore, mice lacking adiponectin show impaired responses to TZDs [96]. Ligand activation of PPAR*γ* in adipocytes is also associated with decreased production of proteins postulated to cause insulin resistance, including TNF-*α* and resistin [97]. Knockouts of TNF, TNF receptors, and resistin show improved insulin sensitivity, consistent with a physiological and/or pathophysiological role for these proteins in modulating insulin responses and systemic metabolism [98, 99].

### 5.2. Skeletal Muscle

The overall improvement of insulin sensitivity which is observed upon glitazone treatment may potentially result from PPAR*γ* activation also in skeletal muscle. Even though PPAR*γ* is expressed at a low level in myofibers of humans and rodents, the net result of skeletal muscle PPAR*γ* activation is potentially relevant, because skeletal muscle is the largest glucose utilizing organ in the body. Mice with genetic deletion of PPAR*γ* in skeletal muscle showed significantly increased whole-body insulin resistance [59, 60, 100], demonstrated either by insulin/glucose tolerance tests or by hyperinsulinemic euglycemic clamp studies, and developed dyslipidaemia, enlarged fat pads, and obesity on high-fat diet [59, 60]. Lipid overload appears to be a primary event in the pathogenesis of insulin resistance, because increased adiposity is observed before the development of overt hyperglycemia or hyperinsulinemia and despite reduced dietary intake [59]. In addition, Hevener et al. [60] postulated that loss of PPAR*γ* resulted in skeletal muscle insulin resistance followed by impaired insulin action in adipose tissue and liver. By contrast, Norris et al. [59] did not observe any change in muscle glucose disposal, whereas hepatic insulin sensitivity was found to be impaired. Regardless of the basis for these conflicting results, it appears that the pharmacological response to TZDs is preserved, at least under some experimental conditions, in mice lacking PPAR*γ* selectively in muscle. Thus, it is unlikely that a direct action on muscle is the primary basis for the clinical effects of PPAR*γ* agonists, again underscoring the importance of adipose tissue as the main mediator of TZD actions [101].

### 5.3. Liver

In experimental models with ablation of white adipose tissue, hepatic PPAR*γ* participates in both fat regulation and glucose homeostasis, and TZD treatment results in lower triglyceride and glucose levels [102]. However, when adipose tissue is normally expressed, the impact of PPAR*γ* in the liver on glucose homeostasis appears to be minimal. Studies in rodents suggest that activation of hepatic PPAR*γ* signaling promotes lipid accumulation in the liver [102], and hepatic expression of PPAR*γ* is elevated in rodent models of diabetes and insulin resistance that exhibit liver steatosis. Treatment of diabetic mice with TZDs promotes hepatic lipid accumulation, and this effect is abolished in liver-specific PPAR*γ*-null mice [90]. However, expression of PPAR*γ* does not appear to be linked to hepatic steatosis in humans [103]. In fact, studies have suggested that TZDs may be beneficial in treating nonalcolholic fatty liver disease (NAFLD) and nonalcoholic steatohepatitis (NASH) in patients with various degrees of adipose tissue accumulation and metabolic abnormalities [104–106]. However, the ability of PPAR*γ* to directly drive hepatic lipid accumulation in humans treated with TZDs is likely outweighed by the more prominent beneficial effects on fatty acid storage in adipose tissue.

### 5.4. Systemic Effects

Circulating levels of free fatty acids (FFAs) are a major determinant of insulin sensitivity [107]. The activated PPAR*γ* receptors modulate the expression of genes involved in lipid metabolism and promote fatty acid uptake and storage in adipose tissue. Several studies have shown that the antidiabetic efficacy of TZDs correlates with their ability to lower circulating FFA levels [107]. PPAR*γ* activation by TZDs has been shown to reduce the amount of circulating FFA in the body via adipocyte differentiation and apoptosis. The number of small adipocytes, which are able to accumulate FFA, increases at the expense of hypertrophied adipocytes that release FFA. The distribution of adipose tissue is changed from visceral sites to subcutaneous parts of the body. Thus, PPAR*γ* activation results in more efficient accumulation of fatty acids in the subcutaneous depot [90]. Pharmacological data indicate that PPAR*γ* activation in adipose tissue may exert coordinated effects on FFA flux (promoting uptake/trapping, whilst simultaneously impairing release) through the regulation of a panel of genes involved in FFA metabolism. Adipocyte lipoprotein lipase expression is upregulated in response to TZD treatment, thereby potentially enhancing release of FFAs from circulating lipoproteins [108]. Simultaneous upregulation of FFA transporters such as CD36 and fatty acid transport protein on the adipocyte surface facilitates their uptake [109]. TZDs may also reduce FFA efflux from adipocytes through enhanced expression of genes that promote their storage in the form of triglycerides (e.g. glycerol kinase directs the synthesis of glycerol-3-phosphate directly from glycerol; PEPCK permits the utilization of pyruvate to form the glycerol backbone for triglyceride synthesis) [110, 111]. Coordinated regulation of these pathways ensures that FFAs are stored appropriately in adipose tissue, and not “ectopically” in other sites such as liver and skeletal muscle where they are capable of inducing “lipotoxicity.” 

As expected with PPAR*γ* activation, a reduction in plasma FFAs is a consistent observation across many large-scale TZDs clinical trials [112]. This reduction in plasma FFAs also provides a potential mechanism to improve insulin sensitivity in the liver and periphery, as well as reducing lipotoxicity in the pancreatic *β*-cell and improving insulin secretory function. Accordingly, TZD-induced decreases in NEFA correlate with improvements in both muscle and hepatic insulin sensitivity in patients with type 2 diabetes [113]. A study in PPAR*γ* (−/+) mice showed that PPAR*γ* indirectly protects pancreatic islets from lipotoxicity by regulating triglyceride partitioning among tissues (reducing net influx of NEFA into the islets) and that TZDs can restore insulin secretion impaired by lipotoxicity [114]. It is possible that *β*-cell protective effects of TZDs may also be mediated indirectly through reduced *β*-cell stress resulting from the amelioration of insulin resistance. However, based on studies in isolated human islets, there is also evidence that PPAR*γ* activation can have direct effects on *β*-cell function [115, 116].

## 6. Conclusions

PPAR*γ* has emerged as a key regulator of adipocyte and macrophage function in adipose tissue. Direct effects of PPAR*γ* activation on adipose tissue lipid metabolism and endocrine function may be linked with secondary benefits in liver and muscle lipid metabolism and insulin signalling and suggest that PPAR*γ* is an important target for pharmacotherapy to tackle the metabolic syndrome and obesity-related insulin resistance. Furthermore, activation of PPAR*γ* in adipose tissue may also have positive effects on pancreatic *β*-cell function and help to minimize the aggravated paracrine relationship between adipocytes and macrophages seen in obesity. Thus, adipose PPAR*γ* appears to be an essential mediator for the maintenance of whole body insulin sensitivity: protects nonadipose tissues against lipid overload and guarantees appropriate production of adipokines, such as adiponectin and leptin from adipocytes. PPAR*γ* ligands promote the restoration of normal levels of adipose-derived substances, including FFA, TNF-*α*, leptin, adiponectin, and PAI-1, and reverse major defects of the insulin resistance syndrome due to their important effects on inhibition of atherosclerosis, improvement of endothelial cell function, and attenuation of low-grade inflammation.
